# Outcome-based education: evaluation, implementation and faculty development

**DOI:** 10.15694/mep.2020.000121.1

**Published:** 2020-06-16

**Authors:** Shazia Iqbal, Ian Willis, Turky H. Almigbal, Abdullah Aldahmash, Samer Rastam

**Affiliations:** 1 Alfarabi College of Medicine Riyadh KSA; 2 University of Liverpool UK; 3 King Saud University Riyadh KSA

**Keywords:** Outcome-based education, Evaluation of Outcome-based education, implementation of integrated curriculum, Faculty development in medical education, SPICES Model integrated curriculum evaluation, Assessing implementation of medical education

## Abstract

This article was migrated. The article was marked as recommended.

Developments in Outcome-based education (OBE) and innovative shifts in its pedagogical approaches have reshaped the learning environment of curricula at medical schools. This instructional design has gained popularity due to its authenticity and systematic approach. However, this needs organized supervision and faculty training in order to achieve the desired goals for the program.

**Aim:** This article examines the evaluation of OBE at a private medical school in Saudi Arabia. It describes the curriculum review process and the characteristics of the curriculum reviewers involved. It evaluates the curriculum using Harden’s OBE implementation inventory. OBE reviewers’ satisfaction about OBE implementation was evaluated using the OBE inventory.

**Results:** This analysis shows our institutional profile to be similar to the ‘transition to beavers’ symbol in Harden’s representation. At the program level, the study identifies gaps and suggests suitable recommendations to enhance the enactment of OBE.

**Conclusion:** We strongly encourage medical educators to apply the nine components of the OBE implementation inventory to evaluate their level of implementation of OBE. To further build up this model, the authors propose a mnemonic “ADAPTIVE Species” as an instructional prompt to develop these qualities in medical faculty. “ADAPTIVE Species” stands for Assertive, Developer, Assessors, Prime-movers, Transparent, Innovators, Vigilant, Evaluators, and Selectors.

## Introduction

Developments in Outcome-based education (OBE) and innovative shifts in its pedagogical approaches have reshaped the learning environment of curricula at medical schools. This instructional design has gained popularity due to its authenticity and systematic approach ( Rubin and Franchi-Christopher, 2002) This evolving paradigm and evidence-based medical practices in the health care system are provoking a continuous review of curricula and specific learning outcomes. However, OBE implementation needs organized supervision and faculty training in order to achieve the desired goals for the program ( Gruppen, 2012).

The OBE approach involves not only set of specific learning outcomes but it demands successful implementation ( Harden, 2007a, 2009). Effective evaluation requires a set of parameters that serve as a guide to assess the degree of OBE curriculum implementation. Also, there is a great concern to ensure that faculty are able to recognize the importance of OBE and sufficiently skilled to ensure its effective application.

In medical education, there are different evaluation tools to gauge the implementation of OBE; for instance, course reports appraisals, self-study evaluations, program annual reports, student surveys, and external and internal reviews. Additionally, there are models that help medical educators to map the curriculum and to ascertain the progress of implementation of OBE throughout the program in terms of depth, scope, value, and proficiency ( Harden, 2007a, 2007b).

There is an established instrument, the ‘Outcome-based Education implementation inventory’, introduced in 2007 at the University of Dundee by Professor Harden ( Harden, 2007b). The nine components in this model are sufficiently comprehensive and reliable to scrutinize the extent of implementation of OBE and to guide the areas of reform by detecting gaps in the curriculum.

It is important to carry out studies that investigate OBE implementation in diverse learning environments and educational cultures. There are few studies that explore the role of faculty and faculty development programs that train faculty in OBE evaluation and implementation. Additionally, there is lack of evidence to develop faculty development strategies by identifying the loopholes in OBE ( Steinert *et al.*, 2016).

This article examines the application of OBE at a private medical school in Saudi Arabia. It highlights a pragmatic approach to apply the SPICES model in the undergraduate medical curriculum. The SPICES term is abbreviated for; student-centered, problem-based, integrated, community-based, elective and systematic approach. It describes the curriculum review process and the characteristics of the curriculum reviewers involved. In addition, it evaluates the curriculum using Harden’s Outcome-based Education implementation inventory ( Harden, 2007b).

At the program level, the study explores the extent of application of OBE in the MBBS undergraduate curriculum, identifies gaps and suggests suitable recommendations to enhance the enactment of OBE. Furthermore, it assists with faculty development strategies by proposing a model in the form of mnemonic “ADAPTATION Species” to be utilized for faculty development in order to enhance OBE. These suggestions can be generalized to similar OBE programs and educational cultures to support OBE implementation in comparable contexts.

### Implementation of Outcome-based education

In Saudi Arabia, Alfarabi College of Medicine in Riyadh provides a Bachelor’s Degree in Medicine and Surgery (MBBS). The program utilizes OBE and its design is based on the SPICES model ( Harden, Sowden and Dunn, 1984). This conveys a set of core knowledge, skills, and behaviors that are expected to be achieved by the medical graduates through the specific learning outcomes. Significant progress has been made in implementing the OBE and SPICES models at Alfarabi College of Medicine. In the MBBS curriculum, program learning outcomes have been specified in all courses.

The curriculum integrates basic and clinical sciences and focuses on acquiring knowledge through a problem-based learning and student-centered learning approach ( Neville, 2009). With this approach undergraduates develop the ability to seek and address societal, and community issues in the healthcare system ( Hmelo-Silver, 2004). In addition, this framework brings medical students closer to patients as early as possible in the MBBS program ( Preeti, Ashish and Shriram, 2013).

The curriculum is reviewed externally by the Centre for Medical Education CenMEDIC Committee and its framework is considered similar to the CanMEDs Framework that was established by the Canadian Royal College of Physicians and Surgeons in 1996 ( Shadid *et al.,* 2019). Also, it is based on the World Federation for Medical Education (WFME) global standards for basic and quality medical education. In short, this structure provides the students with a strong foundation, which is essential for competent physicians to work in their own diverse cultures and follow regional laws ( Cheng *et al.,* 2014). There is a need to ensure the employment of this curriculum meets international standards, without losing the local perspective.

### The curriculum management committee

The Curriculum Management Committee (CMC) is responsible for the development and reforms of the MBBS curriculum. During regular CMC meetings, the Medical Education Unit aims to make the learning outcomes explicit and to emphasize the use of the specific learning outcomes as a basis for decisions about curriculum reforms. The Medical Education Unit oversees content mapping and alignment of the learning outcomes with the teaching strategies and assessment. It also carries out regular appraisals of course specifications to build and promote those strategies that can cultivate an exciting and engaging learning environment for medical students.

The Medical Education Unit also provides short courses, workshops and 1:1 support for faculty development. A systematic review has shown that this type of support is important for improving teaching effectiveness in medical education. In the past, most of the focus has been on support for individuals, but it is reported that there is also a need for faculty development that supports change across teaching teams and programs ( Steinert *et al.*, 2016).

Alfarabi’s curriculum had been reviewed in a series of six cycles (over each semester) for the last three years. The course reports and content experts’ recommendations were considered as the main drivers for aligning the pedagogical strategies, assessment and the learning outcomes. Focusing on these elements of curriculum alignment aimed to ensure the implementation of the curriculum.

### The Outcome-based Education implementation inventory

In the Outcome-based Education implementation inventory there are three types of institutions/groups of educators; named as ‘the Ostrich’, ‘the Peacock’ and ‘the Beaver’ ( Harden, 2007b). Eloquently, the author has described the characteristics of each group and their approach in relation to OBE.

The Ostriches are a group of medical educators who do not believe in the use of learning outcomes. This group neither favors the use of learning outcomes in the curriculum nor uses them in their teaching. This attitude is a real risk for the sustainability of programs in the current medical education era, which strongly supports and recognizes the values of OBE. The approach of this cluster of instructors is unlikely to survive in the future.

On the other hand, the peacocks are those faulty who agree to put learning outcomes in the papers and proclaim the value of OBE. However, in practice, they fail to implement and apply OBE. For that reason, the applied OBE does not match the displayed learning outcomes in the papers. So, they are merely showing off for visitors or external reviewers and pretend to be committed to this task.

Finally, there are the beavers who are not only strong advocates for setting learning outcomes in the planned curriculum but are also efficient and dedicated to implementation. They aim to work effectively and their efforts are reflected clearly in the form of an impact on the curriculum reforms. Their values, beliefs, and ability to work as catalysts in education environments can promote and reshape medical schools. Undoubtedly, the future survival and success in the competitive high-stakes healthcare education environment belongs to them ( Harden, 2007b).

## Methods

In order to assess the level of adoption of OBE in the Alfarabi curriculum, we applied the Outcome-based education implementation inventory. A survey was designed on a Google form. This included the nine components of the OBE implementation inventory profile. This form was disseminated through email to the CMC members, including the external reviewers.

These members of CMC were not only content experts but also context specialists for curriculum review. All of them and the external reviewers have more than 10 years of teaching experience and the CMC members hold key positions at the medical school as course directors/course organizers. They were regularly involved in the review process and have a wide range of experience; including teaching, assessing, quality assurance, and medical education.

Thirty participants were sent a Google form survey designed on the five point Likert scale against nine components of the OBE implementation inventory. Participants were required to choose the satisfaction level for the achievements of each component mentioned in the OBE implementation inventory.

They were instructed to use their estimations of satisfaction levels based on their three years of experience involved in the MBBS curriculum review at Alfarabi College of Medicine. Out of 30 participants, 23 responded and filled out the survey. The responses for each factor were used to draw the OBE implementation profile of our institution ( Harden, 2007b). These components were further simplified with a brief description of each section to elaborate on the meanings for participants.

The following are the components of the implementation inventory:

1. Statements of learning outcomes (This dimension reflects the extent to which there is a clear statement of the learning outcomes in courses)
2. Communication with staff/students about the learning outcomes (This aspect is a measure of the extent to which staff and students in an institution are made aware of the existence of an outcome statement and are familiar with it)
3. The educational strategies adopted (The choice and use of teaching methods including lectures, small group work, blended learning, and independent study should reflect the learning outcomes)
4. The learning opportunities available, (the use of new learning technologies, use of simulators, skill labs, technology-enhanced learning, high fidelity simulators, audience response systems, poll systems in lectures)
5. The course content (Consideration of the learning outcomes and the danger of information gap or overload, curriculum congestion, while content mapping)
6. Student progression through the course, (Learning outcomes are usually expressed as the gained competencies expected at the end of an education program)
7. Assessment of students (The summative and formative assessment methods adopted must reflect the agreed learning outcomes and informed decisions taken, as to whether a student has or has not achieved the stated outcomes)
8. The educational environment (The learning outcomes should inform what was seen as a desirable learning environment. For example, if the ability to work as a member of a team is a learning outcome, an educational environment that supports collaborative working was more appropriate than the more typical environment where competition is rewarded)
9. Student selection (In Outcome-based education, the approach was adopted while admitting the students, based on the level of achievement expected of students prior to entry to medical studies in each of the outcome domains such as communication skills, decision making, attitudes, ethics, and practical skills)

## Results/Analysis

Assessing the OBE by utilizing the implementation inventory facilitates evaluation of the operational and planned curriculum. It not only helps to enhance the curriculum but also helps to bring transformation in pedagogical schemes to align the taught content and exit program learning outcomes.

Almost half of reviewers (n 11= 47.8%) strongly agreed (score 5) that we have clear and explicit program learning outcomes matching with course learning outcomes distributed over the six years of the MBBS program as attached in Appendix Figure 3 (a-i). Nearly half of reviewers (n 10= 43.5%), agreed (score 4) that programme learning outcomes had been communicated to students clearly.

Regarding the course content, student progression, assessment methods, and educational environment, the participants were agreed on the following highest percentages (n 11= 47.8%) agreed (score 4), (n 15= 65.2%) agreed (score 4), (n 13= 56.5%) agreed (score 4), (n 11= 47.8%) agreed (score 4).

All most half of reviewers (n 11= 47.8%) strongly agreed (score 5) in educational strategies adopted and learning opportunities components. About the student selection, a low number of participants (n 5= 21.7%) strongly agreed, however, the majority of reviewers (n 6= 26.7%) showed a neutral Score (3) or (n 6= 26.7%) agreed score (4).

## Discussion

### Statements of learning outcomes

**Figure 1.**
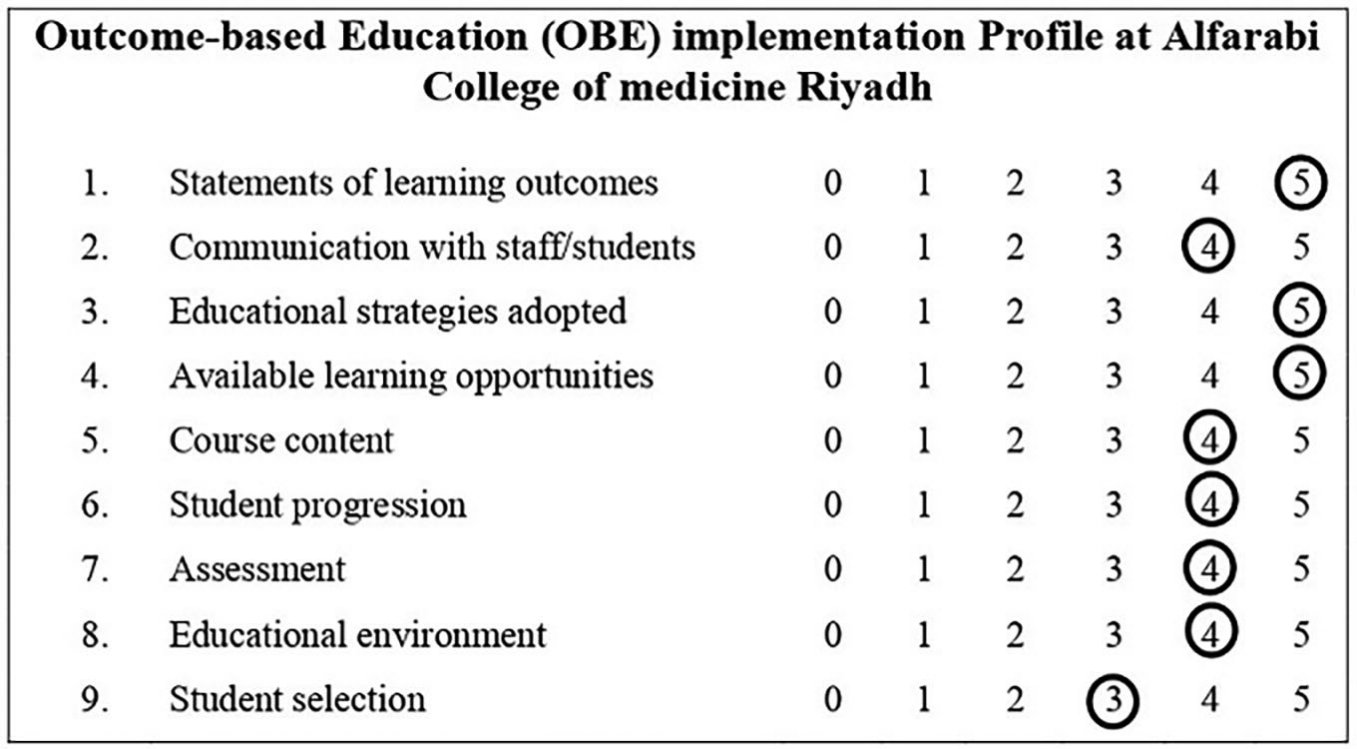
Alfarabi Outcome-based Education (OBE) implementation inventory matching the Harden’s “Transition to the Beaver” profile

The level of agreement in this component demonstrated that the programme is achieving this component to the reasonably satisfactory level. It was indicative of possessing the **Beavers’** profile in Harden’s OBE inventory profile, which is quite inspiring for the programme reviewers as shown in Figure 1.

### Communication with staff/students

This analysis signified the areas of improvement and gave ideas to bridge this gap by setting an effective means of communication about the learning outcomes to students in a more explicit manner. Nearly half of reviewers (n 10= 43.5%, score 4) depicted that we are going in the right direction however need to work hard in order to be ‘the **Beaver’.** Also, we need to inquire further about the reasons for this lack of communication. We must involve the stakeholders (faculty, students, owners, policymakers, medical educators) of the curriculum to diminish this weakness. Any further neglect of this aspect will likely push the institution towards the peacock’s profile, which would be alarming.

### Educational strategies adopted and learning opportunities

As far as these two parameters were concerned the results aligned with the beaver profile. This satisfaction level is encouraging for the organization and faculty to remain confident and progress towards the goal of the institution.

As there is always room for improvement, henceforward, the authors suggested creating more exciting and engaging learning opportunities. This could be augmented by designing instructional methods that ensure interaction between the tutors and students to provoke meaningful learning experiences. The use of novel learning software, practice on trainers (Standardized patients); high fidelity simulators supported by technology-enhanced learning; audience response systems (Poll-everywhere), and artificial intelligence can boost the learners’ interest in the subject.

### Course content, Student progression, Assessment, and Educational environment

Perhaps the most important aspect for our program is to focus on is the content mapping and the monitoring the students’ progress throughout the courses by introducing robust formative assessments. Overall, these scores from the implementation inventory profile indicated that we fell in the category of strugglers to be the beaver as shown in Figure 1. This analysis shaped our institutional profile almost similar to **the transition to beavers.** Significantly, these findings supported the efforts to implement the OBE and to continue the efforts to establish the profile of the beavers.

In addition, the authors suggest that formative feedback, formative assessments, and continuous motivation of students, faculty, and educators throughout the program can build a vigorous team which can considerably boost the OBE employment. Ultimately, it will promote an atmosphere to cultivate the optimal level of medical practitioners and institutions will propagate in the medical profession.

### Student selection

Certainly, the score in these components was a true reflection of the prevailing situation and demanded most of the stakeholders’ attention. Our findings a real need to improve student selection criteria and are closely aligned with “the peacock tail”. The authors suggest that student selection is a key area to develop robust selection criteria for admission in the MBBS program. In fact, these findings require further research as to the reasons for the reviewers’ opinions. Identifying the failings can help institutions to make plans to combat the gaps and modify the student selection criteria.

Finally, it is suggested that one must not only regard the selection criteria in terms of mark sheets/reports or summative assessments but it is equally crucial to match the selection criteria to the program learning outcomes. For instance, if we require that medical students must demonstrate excellence in ethics, communication skills, and professionalism, then there could be a preliminary assessment of communication skills, professionalism and ethical attributes at the point of entry to the program.

In this study, the researchers determined that in order to be true to the beaver, we need to be as adaptive as the beaver and secure transformation in the educational ecosystem. Faculty and medical educators have to possess the unique survival characteristics of the beaver such as fortitude, grit, and acceptance to change ( Fullan, 2011). Only then we can claim to be the actual beaver, which is a symbol of determination, intuition, and diligence.

Besides, medical educators and institutions ought to instill the spirit of real beavers and implement the OBE curriculum reforms. Implementing the innovations in OBE and enhancing the educational environment will assist t institutions to flourish and strengthen the capability to keep pace with international standards of medical education.

Faculty development: “To Be beavers; Be adaptive”

**Figure 2.**
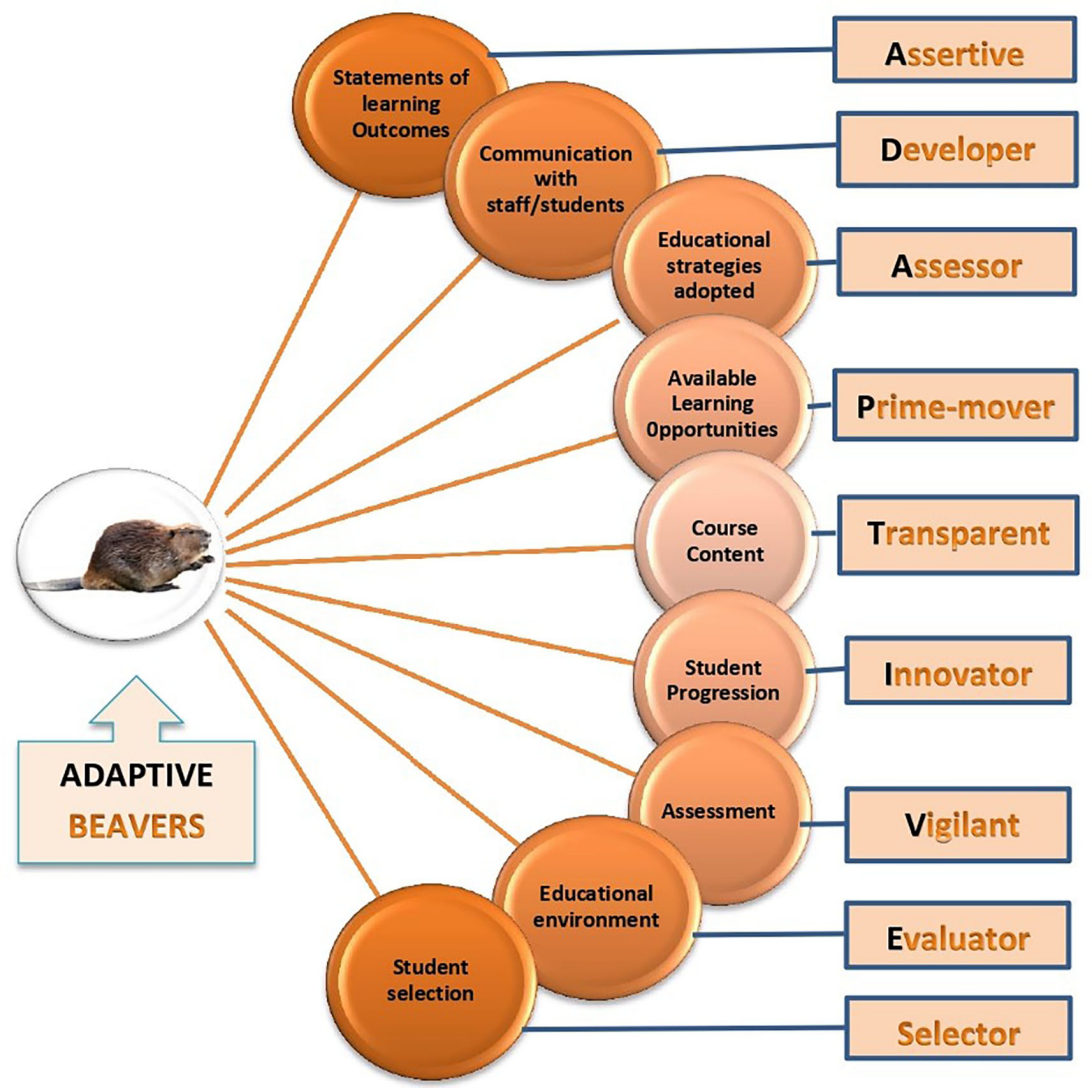
Nine characteristics in medical educators can augment nine components of “outcome-based Education (OBE implementation inventory)” to enhance OBE & To “Be the Beavers; Be Adaptive Species”

The authors of this article strongly encourage medical educators to apply the nine components of the OBE implementation inventory to evaluate their level of implementation of OBE. To further build up this model, the authors propose a mnemonic **“ADAPTIVE Species”** as an instructional prompt to develop these qualities in medical faculty as shown in Figure 2.

It highlights that medical school stakeholders need to be **A**ssertive in the planning and application of program learning outcomes in the program. They must **D**evelop the vision to enhance effective and efficient communication of program learning outcomes among students and faculty to bridge the communication gap ( Yardley, Irvine and Lefroy, 2013). As **A**ssessors, they have to devise educational strategies which ensure constructive alignment in OBE curricula (Davis *et al.* , 2007).

Furthermore, faculty members have to be determined and committed to cultivating favourable and exciting developments corresponding with OBE. They must serve as key **P**rime movers and motivators to enhance the learning environment and keep the learner engaged in the process of learning ( Artino, Holmboe and Durning, 2012). While establishing constructive alignment and revisiting the course content; medical educators are encouraged to be **T**ransparent and accountable. Medical institutions can support faculty development by providing opportunities for frequent training workshops and by creating a climate that facilitates open discussion and learning.

In short, as **I**nnovators, medical schools ought to engage in those pedagogical transformations that can provide effective feedback and ensure students’ progress throughout the program (Thomas *et al*., 2016). At the same time, we need to be very **V**igilant in assessment planning to ensure constructive configuration with learning outcomes and teaching methods especially in clinical teaching ( Barrow, McKimm and Samarasekera, 2010).

As curriculum **E**valuators, our approach must be pragmatic and logical to ensure a vigorous educational atmosphere and we must propose strategies which underpin the idea of active learning ( Murdoch-Eaton and Whittle, 2012). While making decisions about the selection of potential medical students, as **S**electors our approach should be holistic and realistic ( Donnon, Paolucci and Violato, 2007). While choosing for entry in the MBBS program, consideration must be given to effective communication skills, professionalism, and ethical values as they are core constituents of OBE ( Howe *et al.*, 2004).

## Conclusion

OBE is demanding in current medical education and requires a vigorous evaluation strategy to assess its implementation. In order to evaluate the application of OBE and identify the gaps, medical educators must design and follow the guidelines. This article utilized the OBE implementation inventory as an OBE evaluation tool and considered it a highly effective instrument to assess the application of OBE at program level.

The representation of faculty satisfaction regarding the employment of OBE is based on the implementation inventory profile. The final shape depicts the operational state of OBE and assists us to identify the areas of improvement. This is promising and encouraging for the institution in that we stand in “the transition to the beaver” and foresee our institution to soon to be the beavers. In order to reach that point, the suggested model of “ADAPTION Species” provides a comprehensive approach to support the development of these characteristics in faculty in order to augment the nine components of the OBE inventory.

To sum up, institutions must promote faculty development courses or workshops to train their faculty in improving the skills and attitudes needed to implement OBE. Apart from medical education, this proposed instructional mnemonic can be generalized to all those programs in higher education which are grounded in an outcome-based curriculum.

## Take Home Messages

• Outcome-based education (OBE) is demanding in current medical education and requires vigorous evaluation strategy.
• OBE evaluation helps to identify gaps and areas of faculty development.
• The OBE implementation inventory proposed by Prof. Harden can serve as an evaluation tool and is considered to be a highly effective instrument to assess the implementation of OBE.
• There is a need for faculty development through workshops and refresher short courses to implement OBE.
• Faculty developers must design and follow the guidelines of OBE evaluation tools.
• The authors propose a mnemonic “ADAPTIVE Species” (as an instructional prompt) to develop the required qualities in faculty

## Notes On Contributors

This study was supported by Research Unit, Alfarabi College of medicine Riyadh. The author of this research is thankful for Dr. Ian Willis, Dr. Samer Rastam & Dr. Abdullah M. Aldahmash for reviewing this article and Dr. Turky H. Almigbal for being coauthors of this article.

**Dr. Shazia Iqbal** is working as Director Medical Education at Alfarabi College of Medicine, Riyadh, Saudi Arabia. She assists in the development and review of OBE/ integrated curriculum at medical institutions with a special interest in pedagogical techniques and innovative educational technologies.

**Dr. Ian Willis** supervises internationalization theses on the University of Liverpool’s online Professional Doctorate in Higher Education (EdD) UK. He is a principal fellow of the Higher Education Academy UK (Advance HE) and formerly the Head of the Educational Development Division at UoL.

**Dr. Turky** **H. Almigbal** is assistant professor, family and community medicine department, College of Medicine, King Saud University, Riyadh. His has keen interest in curriculum development and reviews.

**Dr. Abdullah M. Aldahmash** is Dean of Alfarabi College of Medicine Riyadh. He is founder and director of Stem Cell Unit, King Saud University, Riyadh. He has profound interest in faculty development and innovation in medical education.

**Dr. Samer Rastam** is supervisor Research Unit, Alfarabi College of medicine Riyadh. He has keen interest in integrated curriculum and content mapping. He is expert in quantitative data analysis.

## Appendices

Figure 3 (a-i): Summary of Alfarabi Outcome-based Education (OBE) implementation inventory Score
