# Supplementary material for: Outcome-based education: evaluation, implementation and faculty development
Source: MedEdPublish (2016). 2020 Jun 16;9:121. [Version 1] doi: 10.15694/mep.2020.000121.1 (PMC10702664; doi:10.15694/mep.2020.000121.1)
Supplement: Peer review history

# Reviewer report: Reviewer response for version 1

**DOI:** 10.21956/mep.20004.r30484

**Published:** 2020-08-14

**Authors:** Megan Anakin

**Affiliations:** 1 University of Otago

This review has been migrated. The reviewer awarded 3 stars out of 5 Thank you for inviting me to review your article, Shazia. I read your article with interest. My medical school is undertaking a curriculum review process so I was curious to see how you evaluated the programme at your medical school. The introduction describes outcomes-based education (OBE) and states the evaluation approach used in your study. To enhance understanding of your evaluation approach, please consider describing the nine components of the evaluation model and the mnemonic ‘ADAPTATION Species’ so the reader can better understand how you used it to evaluate your programme. The context of the study is well described and it includes a description of the OBE programme, curriculum management committee, and an OBE implementation inventory. To enhance the introduction for the reader, please consider moving the aim of your study to the end of this section and explain the relevance of these last three study context components and how they relate to the aim of your study. Please consider including the survey that was used and adding a paragraph to the methods section that explains how you analysed the data you collected from participants using the implementation inventory. Please consider moving the first two sentences of the results section to the beginning of the discussion section because they are statements that interpret the results before the results of the study have been presented. Please consider providing demographic information about the participants/reviewers such as gender, ethnicity, role in the medical programme, professional background/expertise, and length of time as a clinical educator. Please explain how scores in the inventory profile were interpreted to correspond to the Beaver profile in Harden’s OBE inventory in the methods section and please consider reporting this as a result in the results section. This additional information and change to the article will help the reader appreciate the significance of the Beaver profile of staff at your university and the implications for curriculum review that readers can learn from if they have staff with Beaver profiles at their medical schools. To enhance the presentation of the mnemonic ‘ADAPTATION Species’ in the discussion, the authors may with to discuss how they might use and evaluate this approach with their staff in a future study. Missing from the discussion is a paragraph reflecting on the strengths and limitations of the study design including a statement about how the evaluation model used allowed particular features of the curriculum to be studied and how sample of participants had an impact on the interpretation of the quantitative results including generalisability, representativeness, and applicability. For example, as a reader I am wondering about the views of other stakeholders such as students and patients. I would like to encourage the authors to submit a revised version of their article to strengthen their message to readers because the authors have taken a productive approach to reviewing curriculum in their study.

Reviewer Expertise:

NA

No decision status is available

# Reviewer report: Reviewer response for version 1

**DOI:** 10.21956/mep.20004.r30482

**Published:** 2020-08-12

**Authors:** Sateesh Babu Arja

**Affiliations:** 1 Avalon University School of Medicine

This review has been migrated. The reviewer awarded 4 stars out of 5 I read this article with much interest and thoroughly enjoyed while reading this manuscript. I must commend the curriculum committee and Medical Education Unit for implementing the outcome-based education and for providing faculty development activities. The framework designed for the inventory matching of implementation of outcome-based education seems appropriate. But as Professor Gibbs pointed out, the methods could have been more appropriate if there is a separation of responses or results between internal reviewers and external reviewers. Thank you.

Reviewer Expertise:

NA

No decision status is available

# Reviewer report: Reviewer response for version 1

**DOI:** 10.21956/mep.20004.r30483

**Published:** 2020-08-04

**Authors:** Trevor Gibbs

**Affiliations:** 1 AMEE

This review has been migrated. The reviewer awarded 3 stars out of 5 I enjoyed reading this paper although I feel that there could be a lot of improvement in the research methodology. It seems to me that the questions related to the evluation of OBE was biased towards the positive, given that the evaluators were evaluating their own work. I recognise that the authors suggested that external reviewers were involved, so this situation could have been improved by having those reviewers evaluations taken out and discussed separately. I also feel that, in this piece of research, the users of the produce i.e. the students, would provide valuable feedback.I enjoyed reading the use of ADAPTIVE approaches and that I feel reflects a very useful approach.Clearly the faculty and its curriculum committee have done a good job in developing this OBE curriculum and if they had re-structured their research approach more effectively, then I would have been inclined to give it a higher star rating.It is well worthwhile running another evaluation exercise with a wider, more diverse, less-biased evaluation team.

Reviewer Expertise:

NA

No decision status is available

# Reviewer report: Reviewer response for version 1

**DOI:** 10.21956/mep.20004.r30487

**Published:** 2020-07-27

**Authors:** P Ravi Shankar

**Affiliations:** 1 American International Medical University

This review has been migrated. The reviewer awarded 4 stars out of 5 The authors provide a detailed description of the outcomes-based curriculum at their university. They have evaluated the implantation of outcomes-based education (OBE) at the institution using the OBE implementation inventory. The ADAPTIVE species mnemonic proposed is interesting and informative. The authors can provide more background information about the college, the educational program followed, the student selection procedure and characteristics of the admitted students. This will help readers make better sense of the study findings. Among the 23 respondents it will be interesting to know how many were external reviewers? Were there any differences in perception between external reviewers and members of the CMC? Figure 2 is interesting and informative. One of the limitations could be those surveyed were also involved in developing and monitoring the OBE curriculum. The key words are very long and could be shortened. The quality of written English is good. In certain places however, I am of the opinion that some of the words used can be replaced by more appropriate ones. The article will be of broad interest to educators especially those involved with outcomes or competency-based curricula.

Reviewer Expertise:

NA

No decision status is available

# Reviewer report: Reviewer response for version 1

**DOI:** 10.21956/mep.20004.r30481

**Published:** 2020-06-18

**Authors:** J Hartmark-Hill

**Affiliations:** 1 University of Arizona College of Medicine-Phoenix

This review has been migrated. The reviewer awarded 1 stars out of 5 As medical education transitions to outcomes- based and competency-based education, the need for expanded, evidence-based program evaluation measures and iterative faculty development is an area of importance to curricular leaders.The authors provide adequate information in the background to understand the need for this research and subsequent study.However, research question(s)/hypotheses are not clearly developed, and application of an attitudinal survey of faculty toward efficacy of various curricular elements does not address the original premises.While presented as experienced content experts, participants in the survey were course directors and others who had been responsible for creation of the curriculum. Therefore, their own satisfaction with such is subject to bias and blindspots. One example of a more helpful approach would have been a knowledge-based survey regarding levels of implementation (and measures of success) across the curriculum (with comparison to actual status of such). A disconnect between the two could support a case for the need for further faculty development. No demographic data or methodology for statistical analysis of groups was provided. Only 30 faculty were surveyed at a single point in time, and of those, less than 80% of those surveyed responded, likely further skewing results.While the conceptual model presented is of interest for further study, conclusions cannot be made regarding either the data as supporting programmatic success, the need for specific faculty development or generalizability to other institutions.

Reviewer Expertise:

NA

No decision status is available

# Reviewer report: Reviewer response for version 1

**DOI:** 10.21956/mep.20004.r30486

**Published:** 2020-06-17

**Authors:** Felix Silwimba

**Affiliations:** 1 University of Lusaka

This review has been migrated. The reviewer awarded 5 stars out of 5 this is a clearly written easy to follow study report and motivating . I copy the idea adaptive species. I will introduce it in my medical school

Reviewer Expertise:

NA

No decision status is available

# Reviewer report: Reviewer response for version 1

**DOI:** 10.21956/mep.20004.r30485

**Published:** 2020-06-17

**Authors:** Shahzad Ahmad

**Affiliations:** 1 Fatima Memorial medical and dental college Lahore Pakistan

This review has been migrated. The reviewer awarded 5 stars out of 5 I enjoyed reading this paper with great interest. Article is well structured, clear and the idea of evaluating the OBE curriculum is well justified. Author’s recommendations are thought provoking and prompt the medical educators to bring further ideas for OBE curriculum evaluation. At institutional level, medical education demands a structured framework for faculty development and this manuscript has highlighted those essential components and faculty characteristics required in operational OBE curriculum. Especially, the idea of “ADAPTIVE Species” is well fitted in current situation where institutions are moving towards innovative approach of learning and teaching through maximising use of technology.I shall strongly recommend applying this method of evaluating OBE curriculum at instructions.

Reviewer Expertise:

NA

No decision status is available
